# Pneumococcal carriage and changes in serotype distribution post- PCV13 introduction in children in Matiari, Pakistan

**DOI:** 10.1016/j.vaccine.2024.126238

**Published:** 2024-10-03

**Authors:** Izn Iqbal, Shahira Shahid, Samiah Kanwar, Furqan Kabir, Fayaz Umrani, Sheraz Ahmed, Waqasuddin Khan, Muhammad Farrukh Qazi, Fatima Aziz, Sahrish Muneer, Adil Kalam, Aneeta Hotwani, Junaid Mehmood, Abdul Khalique Qureshi, Zahra Hasan, Sadia Shakoor, Shaper Mirza, Lesley McGee, Stephanie W. Lo, Narender Kumar, Iqbal Azam, Stephen D. Bentley, Fyezah Jehan, Muhammad Imran Nisar

**Affiliations:** a Department of Pediatrics and Child Health, Aga Khan University, Karachi, Pakistan; b Infectious Diseases Research Laboratory (IDRL), Department of Pediatrics and Child Health, Aga Khan University, Karachi, Pakistan; c Department of Community Health Sciences, Aga Khan University, Karachi, Pakistan; d Department of Pathology and Laboratory Medicine, Aga Khan University, Karachi, Pakistan; e Syed Babar Ali School of Science and Engineering, Lahore University of Management Sciences, Lahore, Pakistan; f Division of Bacterial Diseases, Centers for Disease Control and Prevention, Atlanta, GA, USA; g Parasites and Microbes, Wellcome Sanger Institute, Hinxton, UK

**Keywords:** *Streptococcus pneumoniae*, Pneumococcal carriage, Pneumococcal conjugate vaccine, Bacterial genomics, Genomic epidemiology

## Abstract

**Background:**

In early 2021, the 10-valent Pneumococcal conjugate vaccine (PCV10) was replaced with 13-valent (PCV13) by the federal directorate of immunization (FDI), Pakistan. We assessed the impact of a higher valent vaccine, PCV13, on the serotype distribution of nasopharyngeal carriage in rural Pakistan.

**Methods:**

Children <2 years were randomly selected from two rural union councils of Matiari, Sindh in Pakistan between September–October,2022. Clinical, sociodemographic and vaccination histories were recorded. Nasopharyngeal swabs were collected and processed at Infectious Disease Research Laboratory, Aga Khan University, Karachi. Whole genome sequencing was performed on the culture positive isolates.

**Results:**

Of the 200 children enrolled, pneumococcus was detected in 140(70 %) isolates. Majority of age-eligible children (60.1 %,110/183) received 3 PCV13 doses. PCV10 carriage declined from 13.2 %(78/590) in 2017/18 to 7.2 % (10/140) in 2022, additional PCV13 serotypes (3, 6A/6C and 19A) decreased from 18.5 %(109/590) to 11.4 %(16/140) while non-PCV13 serotypes increased from 68.3 %(403/590) to 81.4 %(114/140). There were 88.5 %(*n* = 124), 80.7 %(*n* = 113), 55.0 %(*n* = 77), and 46.0 %(*n* = 65) isolates predicted to be resistant to cotrimoxazole, penicillin(meningitis cut-off), tetracycline, and erythromycin respectively.

**Conclusion:**

Replacing PCV10 with PCV13 rapidly decreased prevalence of PCV13 carriage among vaccinated children in Matiari, Pakistan. Vaccine-driven selection pressure may have been responsible for the increase of non-PCV13 serotypes.

## Highlights

• Children <2 had nasopharyngeal swabs whole-genome sequenced.
• PCV13 switch reduced PCV10 serotypes from 13.2 % to 7.2 % by 2022.
• Non-PCV13 serotypes rose from 68.3 % in 2017/18 to 81.4 % in 2022.
• High resistance for cotrimoxazole(88.5 %), penicillin(80.7 %), tetracycline(55.0 %).

## 1 Introduction

*Streptococcus pneumoniae* (pneumococcus) is a leading cause of morbidity and mortality in children under 5 years of age with 9,180,000 episodes of invasive disease and 318,000 associated deaths worldwide [1]. Pakistan was estimated to account for 5 % (14,400 deaths, 95 % CI 9700–17,000) of the global pneumococcal deaths. Colonization is the prerequisite for progression to serious invasive diseases such as meningitis, septicemia, and pneumonia, as well as milder but more common illnesses such as sinusitis and otitis media [2]. Pneumococcus has over 100 known serotypes and is a common colonizer of the human nasopharynx, particularly in children younger than 5 years [3]. Pneumococcal conjugate vaccine (PCV) induces a serotype-specific immune response against many of the serotypes associated with high invasiveness and antibiotic resistance and has led to a significant reduction in the burden of under-5 childhood mortality and disease severity [4].

In a survey conducted in Matiari, Pakistan, before the introduction of the pneumococcal conjugate vaccine (PCV) in 2012, the pneumococcal carriage in children younger than 5 years of age was 79.5 % (179/225). Among these children, 33.5 % (60/179) carried a PCV10 serotype, and 53.1 % (95/179) carried PCV13 serotypes [5]. Approximately, 61 % (47/76) of the pediatric invasive pneumococcal disease (IPD) episodes during this period were caused by PCV10 serotypes and 63 % (48/76) were caused by PCV13 serotypes [6]. In 2012, Pakistan introduced the ten valent pneumococcal conjugate PCV10 in its Expanded Program on Immunization (EPI) with support from 10.13039/100001125Gavi, the Vaccine Alliance [7]. Following PCV10 introduction, vaccine effectiveness was estimated to be 81.9 % (95 % CI −55.7 to 97.9) against IPD in a case-control study conducted at 16 hospitals in Sindh Province between 2013 and 2017 [8]. In a repeat survey conducted in Matiari, Pakistan, the vaccine uptake rate in 2017–18 was 68.4 %. A decline in PCV10 serotypes 23F, 6B, 9V/9A, and 19F was observed, along with a concomitant emergence of serotype 19 A, a serotype included in PCV13 but not PCV10 [9]. In early 2021, Pakistan replaced PCV10 with PCV13 as a 3 + 0 schedule in the national immunization program.

In this study, we investigated the impact of the switch from PCV10 to PCV13 on the prevalent serotypes in nasopharyngeal carriage of children under 2 years of age in rural Matiari, Pakistan. In addition, we describe the antibiotic resistance patterns in the post-PCV13 period.

## 2 Methods

### 2.1 Study design and participants

We carried out a cross-sectional survey from September to October 2022 in two Union Councils (Khyber and Shah Alam Shah Jee Wasi) of a rural district Matiari in Pakistan. These councils are sites where surveillance activities are carried out by Aga Khan University, Pakistan. We have previously performed both pre- and post-PCV10 introduction carriage surveys in these union council [5,9,10]. In these surveys, serotyping was performed using multiplex PCR. Details regarding the study methods have been published previously [11]. Briefly, a background demographic surveillance system (DSS) in the area provided a framework for selecting age-eligible children through simple random sampling from an available line-listing. Trained community health workers performed household visits and enrolled all children less than two years of age residing in the study area whose parents provided consent. Each child was only enrolled once in the study. Vaccination history of the child was collected as a combination of caregiver reported or/and card verified (where available). Children with gross nose and throat abnormalities preventing swab collection or with a serious illness requiring hospitalization, as identified by the CHWs, were excluded from the study**.**

Data on household demographics, recent clinical history (including hospitalization and outpatient visits), vaccination history and exposure to household smoke and indoor air pollution were collected by trained study personnel during household visits. A brief clinical exam, including measurements of fever, respiratory rate, and observation for chest wall indrawing, was also conducted. A two-day training was conducted for community health workers to standardize data and nasopharyngeal swab collection methods. The questionnaire was translated, and back-translated between English, Urdu, and Sindhi. It was then pre-tested in the field prior to study implementation. The data was collected during severe flooding in Matiari, coinciding with the onset of dengue and malaria outbreaks. In case of any illness identified by the study personnel during household visits, the children were referred to the nearest primary care centers and mobile outreach facilities that were specially set up for flood-affected areas.

### 2.2 Nasopharyngeal swab collection and laboratory procedures

Nasopharyngeal swab specimens were collected and transported at 2–8 °C from the field site to the Infectious Disease Research Laboratory (IDRL) in Karachi within 8 h of collection as per established World Health Organization's (WHO) consensus methods [12]. In the lab, samples were vortexed in skim milk tryptone-glucose-glycerol (STGG) medium for 10–20 s to disperse the organisms, and afterward, they were frozen at −80 °C in an upright position until further processing. For culture (batches of 20–40), samples were thawed, vortexed, and 200 μl of a sample were added to a mixture of 1 ml rabbit serum and 5 ml Todd Hewitt broth with 0.5 % yeast extract, then incubated for 6 h at 37 °C. Following this, one loopful (10 μl) was inoculated onto bilayer sheep blood and colistin-nalidixic-acid-agar and streaked for the isolation of streptococci. After 18–24 h, plates were examined for the appearance of alpha-hemolytic colonies and susceptibility to optochin and bile solubility.

### 2.3 Genomic sequencing and analysis

DNA was extracted from single pneumococcal colonies using QlAamp DNA mini-Kit (Qiagen, Germany) according to the manufacturer's protocol. Quantification of DNA was assessed through Invitrogen Qubit 1× dsDNA HS assay kit (Thermo Scientific, USA) on Qubit Fluorometer. Sequencing libraries were prepared, using NEBNext® Ultra™ II FS DNA Library Prep Kit (New England Biolabs, USA). The quality of libraries was assessed using Agilent TapeStation (Agilent, USA). Finally, individual libraries were pooled in equimolar concentration and sequenced on NextSeq-2000, using P3 (300 cycles) reagent kit (Illumina, USA) (paired end; 150 bp each) at the Infectious Diseases Research Laboratory (IDRL) at Aga Khan University, Pakistan using Illumina NextSeq-2000 instrument.

The Bioinformatic analyses was initiated through Trimmomatic v0.39 to remove adapters and low-quality bases. De novo assembly and annotation were performed on the trimmed reads using SPAdes v3.8.0 and PROKKA v1.14.5, respectively. Sequence types (STs) were assigned using PubMLST based on a seven-loci sequencing typing scheme and serotypes were inferred using SeroBA v1.0.0 [13,14]. STs were further clustered into clonal complex (CC) based on the allelic profile sharing 6 out of 7 loci. Genomes were screened for the presence of resistance conferring genes tetracycline (*tet*), erythromycin (*erm* or *mef*), chloramphenicol (*cat*) and resistance conferring mutations for co-trimoxazole (*folA*, *folP*) in the CDC pneumococcal typing pipeline database [15]. Penicillin binding protein alleles *pbp1A, pbp2B* and *pbp2X* were used to predict penicillin minimum inhibitory concentrations (MIC) (mg/l) and interpreted the MIC using meningitis cut-off based on CLSI M100-ED28:2018 [16]. Penicillin resistance was defined as MIC of ≥0.12 mg/l. Multidrug resistance was defined as predicted resistance to ≥3 antibacterial classes. Isolates were classified into Global Pneumococcal Sequence Clusters (GPSCs) based on Population Partitioning Using Nucleotide K-mers (PopPUNK v2.6.0) [17] and a reference database version 6 available at https://www.pneumogen.net/gps/assigningGPSCs.html. We constructed a maximum-likelihood phylogenetic tree using FastTree v2.5.1 by mapping each pneumococcal genome against the reference genome of *S. pneumoniae* ATCC 700669 [18]. The resulting phylogeny was mid-point rooted using FigTree v1.4.4 and visualized using Microreact along with metadata including GPSC and CC.

### 2.4 Sample size estimation

We calculated the sample size based on an anticipated 50 % decline in PCV13 serotypes from an estimated baseline prevalence of 22 % [9], using a confidence interval of 95 %, power of 80 %. This resulted in a sample size of 216 after adjusting for a 10 % non-response rate.

### 2.5 Statistical analysis

PCV13 carriage was defined as isolation of any of the 13 serotypes included in PCV13 (serotypes 1, 4, 5, 6B, 7F, 9V, 14, 18C, 19F, 23F, 3, 6A and 19A). Due to known cross-protection with 6 A antigen, serotype 6C was included as a PCV13 serotype in addition to the above 13 serotypes [19]. Non-PCV13 carriage was defined as presence of all other pneumococci including the non-encapsulated pneumococci and those that remained unassigned by the bioinformatics pipeline. Carriage prevalence in the current study was compared with a previous survey from the PCV10 period (2017/2018) era [9]. We used the Chi-square test to compare changes in carriage, whereas for the serotype, and antibiotic resistance comparisons with cell frequencies <5, the Fisher's Exact test was used. All analysis was performed using STATA version 15.0.

### 2.6 Ethics

Ethical approval was obtained from Aga Khan University's Ethical Review Committee (AKU-ERC 2022-7347-21,448). A written informed consent was obtained from legal guardians before commencing enrollment.

## 3 Results

### 3.1 Characteristics of the study participants

**Fig. 1:**
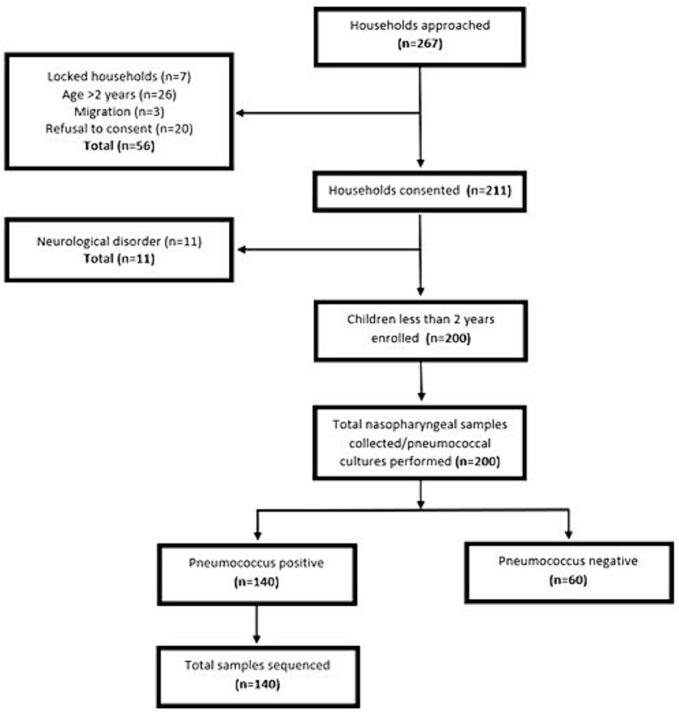
Flowchart describing study population.

We approached a total of 267 households out of which 200 children under 2 years of age were enrolled. The flow of the children into the study is described in Fig. 1. Table 1 describes sociodemographic and clinical and vaccination history of the enrolled children. Mean age of the children was 10.7 ± 6.2 months, 58.5 % were male, majority of the primary caretakers (63.5 %) received no education; median household size was 7; 25.5 % of the children were exposed to environmental tobacco smoke (ETS); natural gas was available in 34.5 % of the household and nearly half of the children were exposed to indoor air pollution by cooking. A history of cough, runny nose and fever in the past two weeks was common. Thirty-two percent had fever at the time of enrollment, 19.5 % had tachypnea and 1.5 % had chest wall indrawing. More than half the age-eligible children 60.1 % (*n* = 110/183) had received all three doses of PCV13, 16.4 % (*n* = 30/183) received 2 doses, 16.9 % (*n* = 31/183) received 1 dose only, and 6.6 % (*n* = 12/183) were unvaccinated.

**Table 1:** Socio-demographic and clinical characteristics of the enrolled children.

Characteristics	Total
	*N* = 200
**Age, months, mean ± SD**	10·7 ± 6·2
0–3 Months	29 (14·5 % %)
4–11 Months	87 (43·5 %)
12–24 Months	84 (42·0 %)
**Gender**	
Male	117 (58·5 %)
Female	83 (41·5 %)
**Mother's age, years, mean ± SD**	30·0 ± 6·4
<20 Years	4 (2·0 %)
20–29 Years	90 (45·0 %)
30–39 Years	82 (41·0 %)
40–49 Years	24 (12·0 %)
**Primary caretaker's education**	
No education	127 (63·5 %)
Primary	48 (24·0 %)
Secondary	20 (10·0 %)
Bachelors	3 (1·5 %)
Master's & above	2 (1·0 %)
**Household size, median(IQR)**	7 (5–10)
**People sleeping in the same room as the child, median (IQR)**	5 (4–7)
**Exposure to environmental tobacco smoke**	51 (25·5 %)
**Exposure to smoke during cooking**	97 (48·5 %)
**Fuel used for cooking**	
Natural gas	69 (34·5 %)
Wood/Paper/Animal dung/Crop residue	131 (65·5 %)
**Child breastfed**	181 (90·5 %)
**Under-weight (Weight for Age** **<** **−2SD)**	105 (52·5 %)
**Stunting (Height for Age** **<** **−2SD)**	103 (51·5 %)
**Wasting (Weight for Height** **<** **−2SD)**	61 (30·5 %)
**PCV13 vaccination status (card-verified/verbal) among age-eligible children** **=** **183**	
Unvaccinated	12 (6·6 %)
One dose	31 (16·9 %)
Two doses	30 (16·4 %)
Three doses	110 (60·1 %)
**Hospitalizations in the previous year**	10 (5·0 %)
**Outpatient visits in past month**	116 (58·0 %)
**Symptoms**	
Cough	67 (33·5 %)
Runny nose	104 (52·0 %)
Difficulty in breathing	25 (12·5 %)
**Signs**	
Temperature	
Hypothermia (≤35 °C)	8 (4·0 %)
Normal	128 (64·0 %)
Hyperthermia (>37·5 °C)	64 (32·0 %)
Tachypnea	39 (19·5 %)
Chest wall indrawing	3 (1·5 %)

Pneumococcus was detected in 140 (70 %) of the isolates which were then whole genome sequenced. The overall carriage rate in children under two years remained unchanged during, before (2017–2018) and after (2022) PCV13 periods (72.8 %, 590/810 vs 70 %, 140/200) respectively (Table 2). PCV10 carriage declined from 13.2 % (78/590) to 7.2 % (10/140) respectively (*p*-value 0.059). The prevalence of PCV13 serotypes (3, 6A/6C and 19A) declined from 18.5 % (109/590) to 11.4 % (16/140) (p-value 0.05). Concurrently, we observed an increase in the proportion of non-PCV13 serotypes from 68.3 % (403/590) to 81.4 % (114/140) (*p*-value 0.001).

**Table 2:** PCV13 and non-PCV13 carriage prevalence over time.

Year	2017/18 survey⁎	2022 survey	p-value
	(*N* = 810)	(N = 200)	
**Number of samples positive for pneumococcus**	**590**	**140**	**0·422**
Prevalence of pneumococcus	72·8 %	70·0 %	
Prevalence of PCV10 serotypes	13·2 % (78/590)	7·2 % (10/140)	0·059
Prevalence of PCV13 serotypes (3, 6A/6C and 19A)	18·5 % (109/590)	11·4 % (16/140)	0·05
Prevalence of non-PCV13 serotypes	68·3 % (403/590)	81·4 % (114/140)	0·001

⁎ Nisar MI, Ahmed S, Jehan F, et al. Direct and indirect effect of 10 valent pneumococcal vaccine on nasopharyngeal carriage in children under 2 years of age in Matiari, Pakistan. Vaccine. Feb 22 2021;39(8):1319–1327. doi:https://doi.org/10.1016/j.vaccine.2020.12.066

### 3.2 Changes in pneumococcal carriage serotypes and GPSCs

**Fig. 2:**
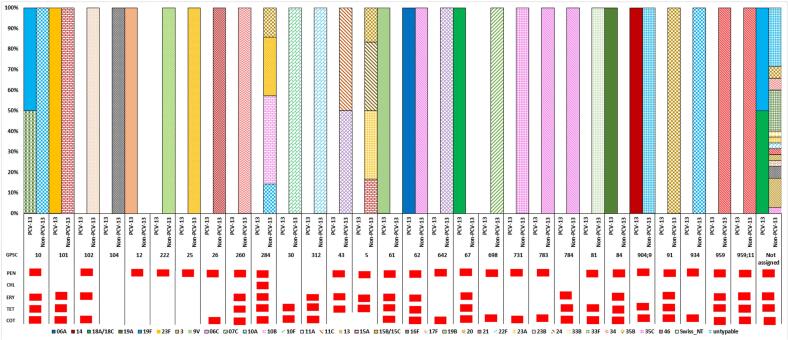
GPSC distribution with associated serotypes in carriage isolates from the post-PCV13 period (2022) in Pakistan (*N* = 140). PEN: Penicillin, CHL: Chloramphenicol, ERY: Erythromycin, TET: Tetracycline, COT: Cotrimoxazole.

Figs. 2 and S1–2 show serotype distribution in the pre-and post- PCV13 period. Overall, 36 serotypes were detected. Of these, the most prevalent serotypes were 10A (*n* = 12), 15B/15C (n = 12), 23B (*n* = 10), 34 (*n* = 9), 33F (*n* = 7), 35B (n = 7), 19A (n = 7), and 13 (*n* = 5), together these serotypes accounted for 49.3 % (69/140) of the total samples. PCV13 serotypes (4, 6B, 14 and 23F, 6A/6C and 19A) declined as compared to the pre-PCV13 survey (*p*-value<0.05). We observed an increase in non-PCV13 serotypes, mainly 10A (n = 12) (p-value = 0.605), 23B (n = 10) (p-value = 0.023), 15B/C (n = 12) (p-value = 0.289), and 34 (n = 9) (p-value = 0.02).

We detected a total of 30 GPSCs in the post-PCV13 period. GPSC102 (serotype 23B; ST16125 and ST4423), GPSC25 (serotype 15B/15C; ST16272 and ST6038), GPSC284 (serotype 15B/15C, 10A, 10B and 35B), GPSC260 (serotype 34) and GPSC10 (serotype 10A, 19A and 19F; ST3135, ST650 and ST319) and were the most common pneumococcal lineages, accounting for 30 % (*n* = 63/140) of the collection. GPSC10 (serotypes 10A, 19A and 19F), GPSC101 (serotypes 23F and 15A) consisted of both PCV13 and non-PCV13 serotypes (Fig. 2). Decrease in PCV13 serotypes 6A/6C and 19A were due to decline in lineages GPSC84, GPSC12 and GPSC62, respectively. The increase in non-PCV13 serotypes were due to GPSC102 (serotype 23B), GPSC25 (serotype 15B/C), GPSC284 (serotype 10A, 10B, 15C and 35B) GPSC260 (Serotypes 34).

**Fig. 3:**
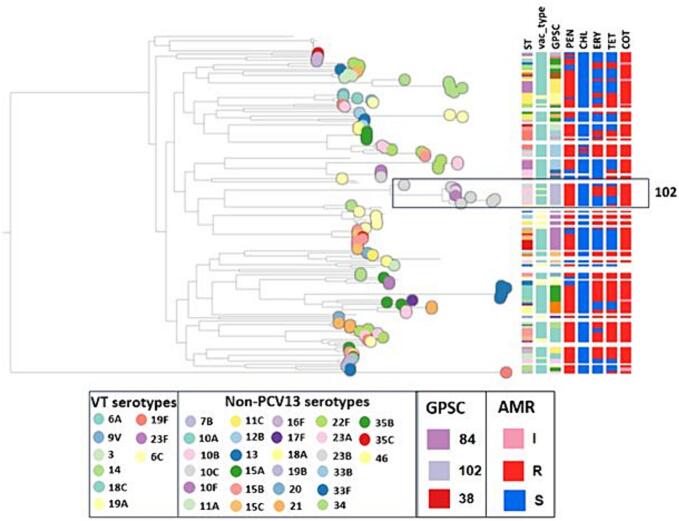
Population structure, serotype and resistance profile of carriage isolates from Pakistan. I: intermediate, R: resistant and S: susceptible.

A phylogenetic tree with the associated metadata is shown in Fig. 3.

Antimicrobial resistance patterns in pneumococcal carriage isolates.

Out of the 140 pneumococcal isolates sequenced, 88.6 % (*n* = 124), 80.7 % (*n* = 113), 55.0 % (*n* = 77), and 46.4 % (*n* = 65) were resistant to cotrimoxazole, penicillin (meningitis cut-off), tetracycline and erythromycin respectively. Mutations in the *folA* gene were detected in all the cotrimoxazole-non-susceptible pneumococci. The majority of the tetracycline-resistant pneumococci expressed *tet(M)* gene(*n* = 74), and of these 84.4 %(n = 65) were resistant to erythromycin and expressed *ermB* (*n* = 35) and *mef* (*n* = 30) genes. A significant increase in erythromycin resistance was detected from 30.8 % (*n* = 182) in the pre-PCV13 survey (2017–18) to 46.4 % (n = 65) post-PCV13 whereas tetracycline resistance increased from 41.9 % (*n* = 247) in the pre-PCV13 survey (2017–18) to 55 % (*n* = 70) post-PCV13. Serotypes 19A, 33F, and 10A among sequence types ST3135 and ST12007 emerged as multidrug resistant. Five GPSCs (101, 104, 30, 312 and 784) had 100 % isolates non-susceptible to penicillin (meningitis cut-off) (Table S1).

## 4 Discussion

We described pneumococcal carriage 18 months after the switch from PCV10 to PCV13 in children under 2 years of age using whole genome sequencing in Matiari, Pakistan. We observed a significant decrease of (38.4 %) in PCV13-specific carriage, despite a modest vaccine coverage of 60.1 % for 3 doses. PCV10 serotypes declined from 13.2 % to 7.2 % which was attributable to decline in serotypes 4, 6B, 23F and 14. The overall carriage rate of 70 % was restored by replacement with non-PCV13 serotypes, predominantly 10A, 23B, 15B/C and 34. This was consistent with previous literature which reported a decline in PCV13 serotypes in invasive disease and carriage globally in the post-PCV13 period [20]. In our study, the direct PCV13 effect on PCV13 carriage was similar to other low and middle-income countries which demonstrated varying rates of direct PCV13 effect depending on factors such as vaccine coverage, schedule, and time since vaccine introduction. Cambodia experienced a 28.4 % reduction in PCV13 carriage with 47 % coverage for 2 doses, 24 months after PCV13 introduction, whereas Lao People's Democratic Republic (Lao PDR) saw a 36.4 % decline with 90 % coverage for 2 doses after 12 months of PCV13 use [21]. Ghana recorded a 23.8 % reduction in PCV13 serotypes with 99.3 % coverage for 3 and The Gambia achieved a substantial 65.7 % reduction with 100 % coverage for 3 doses five years after vaccine introduction [22].

Based on the data from the PubMLST database (www.pubmlst.org), most of the top ten sequence types found in our study were endemic to Pakistan which were mostly collected from Lahore, (ST16127, ST16272, ST16180, ST16130, and ST16278) and were found in south Asia and Africa (India, Nepal, The Gambia, and South Africa) (ST4423 and ST3135). Genomic analysis revealed a change in the serotype composition of several pneumococcal sequence clusters (GPSCs) in the post-PCV13 period. GPSC10 showed an 80 % reduction in 19F, a 50 % reduction in 19A, and a 60 % increase in non-PCV13 serotype 10A in the post-PCV13 period. Similar serotype changes in GPSC10 lineage were also seen in Argentina and Israel post-PCV13 introduction, indicating its ability to consistently survive and expand under vaccine-selective pressure [23]. GPSC10 has a high propensity to cause invasive disease and multidrug resistance and remains among the top five lineages in high IPD burden countries such as India, Pakistan, and Nepal [24]. In addition, we detected a shift in GPSC5 from 19F, 7B, and 24 in both disease and carriage isolates in the pre-PCV13 period to 23 A, 15 A, and 35B in the post-PCV13 period. Both GPSC10 and GPSC5 have been associated with a high serotype diversity and significant increases in non-PCV13 IPD within these vaccine-type GPSCs globally [25]. GPSC5 has also contributed to the majority of 35B/D IPD burden in South Africa and Malawi, and serotype 19A IPD in Israel in the post-PCV13 period [26]. Similarly, the GPSC43 lineage composition was mostly associated with serotypes 9V and 11A in the pre-PCV13 disease isolates whereas we observed exclusively non-PCV13 type carriage (11A, 11C) in this GPSC post-PCV13. In addition, several lineages which expressed only non-PCV13 serotypes, GPSC642 (11A) and GPSC67 (18A/C), GPSC25 (15B/C), GPSC30 (10F) and GPSC26 (46), were predominant in disease and carriage in both pre- and post-PCV13 periods [27]. PCV13 currently provides protection against only three of the serotypes (19A, 19F and 14) found in the prevalent lineages. In addition, we observed serotype 3 to be persistent in carriage in both the pre- and post-PCV13 periods, consistent with findings from other countries indicating that PCV13 is not as effective against serotype 3. This highlights the need to explore higher-valent vaccines to address the increasing incidence of invasive diseases associated with non-PCV13 serotypes. Higher-valent vaccines such as PCV20 (41.4 %, *n* = 53) and PCV24 (41.4 %, n = 53) and PCV25/IVT-25 (55.4 %, *n* = 73) have a higher coverage of the serotypes observed in our setting.

A majority of the pneumococcal carriage samples in our study were resistant against commonly used antibiotic classes in Pakistan such as cotrimoxazole, penicillin (meningitis cut-off), tetracycline, and erythromycin [28]. This may be attributable to a lack of antimicrobial stewardship and poor adherence to infection control guidelines in Pakistan [29]. Most of the GPSCs were multidrug resistant with GPSC284 being resistant to all five classes of antibiotics where GPSC10 was the multidrug resistant lineage in the pre-PCV13 period [27]. Penicillin resistance observed in our study was higher as compared to the pre-PCV13 period in both carriage (53.3 %) and invasive disease samples (61.9 %) [27]. The increase in erythromycin and tetracycline resistance observed in the post-PCV13 period was mediated by the presence of *tet(M), ermB* and *mef* genes along with the expansion of existing non-vaccine serotypes within a well-established resistant lineage such as GPSC10 and GPSC5.

Our findings demonstrate early changes in the pneumococcal carriage population in a low-income setting after PCV13 introduction. This data allows for comparative analyses of population-level effects of the vaccine. As compared to the previous carriage surveys, which utilized multiplex PCR for serotyping, we performed whole-genome sequencing of carriage isolates. This was a major methodological change, which allowed us to detect a greater diversity of serotypes and understand the pneumococcal lineages behind serotype changes following vaccination. Our study has some limitations. There was a slight decrease in the required sample size due to disruption by floods at the study area. There is a lack of genomic data prior to the introduction of PCV13 in Matiari. The study was conducted in rural Matiari district which may limit generalizability of findings to other regions. Variations in socioeconomic conditions and local vaccination practices may introduce confounding variables. Our sampling time-frame (September to October 2022) might have not fully captured potential seasonal variations in pneumococcal carriage. The high proportion of children with fever at the time of presentation were consistent with the ongoing malaria and dengue outbreaks in these flood affected regions [30].The study reports findings 18 months post-PCV13 introduction, and longer-term observations would provide a more holistic understanding of the direct and indirect vaccine impact.

## 5 Conclusion

In conclusion, the study highlights a significant reduction in PCV13-specific carriage among children under 2 years in rural Pakistan after the transition from PCV10 to PCV13. This decline is coupled with a notable shift towards non-PCV13 serotypes, particularly 10A, 23B, 15B/C, and 34. The observed trends align with global patterns of diminished PCV13 serotype-related invasive disease and carriage in the post-PCV13 period. Changes in Global Pneumococcal Sequence Clusters (GPSCs) and antimicrobial resistance against commonly used antibiotics cotrimoxazole, penicillin (meningitis cut-off), tetracycline, and erythromycin. Contribute to the evolving landscape of pneumococcal dynamics post-vaccination. These findings underscore the importance of ongoing surveillance for informed public health strategies.

## Funding

This study was funded by Wellcome Sanger Institute as part of the Global Pneumococcal Sequencing (GPS) consortium funded by Bill &Melinda Gates Foundation through investment ID # INV003570. Funder had no role in collection, analysis, or interpretation of the data.

## Author contributions

M. I. N., W. K., Z. H., F. J., S.B., S.L., S.M., N.K., L.M., I.A., S·S., and F. K. conceived the project idea and designed the research. M. I. N., I.I., S.A., F.U., F.K., A.H., J.M.,A.K.Q., led overall study implementation. W.K., S. K., M.F.Q., F.A., S.M., A.K., J.M.,A.K.Q and S.Sh., were involved in the investigation, data curation, validation, data analysis, and visualization. All authors were involved in the writing-review and editingof the original manuscript. All authors reviewed and approved the final version of the manuscript.

## CRediT authorship contribution statement

**Izn Iqbal:** Writing – review & editing, Writing – original draft, Visualization, Validation, Supervision, Resources, Project administration, Methodology, Investigation. **Shahira Shahid:** Writing – review & editing, Writing – original draft, Visualization, Software, Formal analysis, Data curation. **Samiah Kanwar:** Writing – review & editing, Writing – original draft, Visualization, Validation, Software, Formal analysis, Data curation. **Furqan Kabir:** Writing – review & editing, Writing – original draft, Visualization, Validation, Supervision, Resources, Project administration, Methodology, Investigation, Formal analysis. **Fayaz Umrani:** Writing – review & editing, Writing – original draft, Visualization, Supervision, Resources, Project administration, Methodology, Investigation, Formal analysis. **Sheraz Ahmed:** Writing – review & editing, Writing – original draft, Visualization, Validation, Supervision, Resources, Project administration, Methodology, Investigation, Formal analysis, Data curation. **Waqasuddin Khan:** Writing – review & editing, Writing – original draft, Visualization, Supervision, Software, Project administration, Methodology, Investigation, Formal analysis, Data curation. **Fatima Aziz:** Writing – review & editing, Writing – original draft, Visualization, Supervision, Project administration, Methodology, Investigation, Data curation. **Sahrish Muneer:** Writing – review & editing, Writing – original draft, Resources, Project administration, Methodology, Investigation, Formal analysis. **Adil Kalam:** Writing – review & editing, Writing – original draft, Visualization, Methodology, Investigation, Formal analysis, Data curation. **Aneeta Hotwani:** Writing – review & editing, Writing – original draft, Visualization, Supervision, Software, Resources, Methodology, Investigation, Funding acquisition, Formal analysis. **Junaid Mehmood:** Writing – review & editing, Writing – original draft, Visualization, Validation, Supervision, Software, Formal analysis, Data curation. **Abdul Khalique Qureshi:** Writing – review & editing, Writing – original draft, Visualization, Validation, Supervision, Software, Formal analysis, Data curation. **Zahra Hasan:** Writing – review & editing, Writing – original draft, Visualization, Supervision, Project administration, Methodology, Investigation, Funding acquisition, Conceptualization. **Sadia Shakoor:** Writing – review & editing, Writing – original draft, Visualization, Validation, Supervision, Resources, Project administration, Methodology, Funding acquisition, Formal analysis, Conceptualization. **Shaper Mirza:** Writing – review & editing, Writing – original draft, Visualization, Resources, Project administration, Methodology, Investigation, Funding acquisition, Conceptualization. **Lesley McGee:** Writing – review & editing, Writing – original draft, Visualization, Resources, Project administration, Methodology, Investigation, Funding acquisition, Formal analysis, Conceptualization. **Stephanie W. Lo:** Writing – review & editing, Writing – original draft, Visualization, Supervision, Software, Resources, Project administration, Methodology, Investigation, Funding acquisition, Formal analysis, Conceptualization. **Narender Kumar:** Writing – review & editing, Writing – original draft, Visualization, Supervision, Resources, Project administration, Methodology, Investigation, Funding acquisition, Formal analysis, Conceptualization. **Iqbal Azam:** Writing – review & editing, Writing – original draft, Visualization, Methodology, Investigation, Formal analysis, Conceptualization. **Stephen D. Bentley:** Writing – review & editing, Writing – original draft, Visualization, Validation, Supervision, Software, Resources, Project administration, Methodology, Investigation, Funding acquisition, Formal analysis, Data curation, Conceptualization. **Fyezah Jehan:** Writing – review & editing, Writing – original draft, Visualization, Validation, Supervision, Software, Resources, Project administration, Methodology, Investigation, Funding acquisition, Formal analysis, Data curation, Conceptualization. **Muhammad Imran Nisar:** Writing – review & editing, Writing – original draft, Visualization, Validation, Software, Formal analysis, Data curation, Supervision, Funding acquisition.

## Declaration of competing interest

The authors declare that they have no known competing financial interests or personal relationships that could have appeared to influence the work reported in this paper.

## Data Availability

Data will be made available on request.

## Appendix A Supplementary data

Supplementary material 1- Serotype distribution in the pre- and post PCV13 period Image 1

Supplementary material 2- Antimicrobial resistance patterns Image 2
