# Supplementary material for: Pneumococcal carriage and changes in serotype distribution post- PCV13 introduction in children in Matiari, Pakistan
Source: Vaccine. 2024 Oct 3;42(23):None. doi: 10.1016/j.vaccine.2024.126238 (PMC11413484; doi:10.1016/j.vaccine.2024.126238)

**Figure S1 Serotype distribution in the pre- and post PCV13 period ^10^**

**Figure 2 Distribution of the ten most prevalent serotypes PCV13 and non-PCV13 serotypes in the pre- and post PCV13 period [9] (***p-value<0.05)


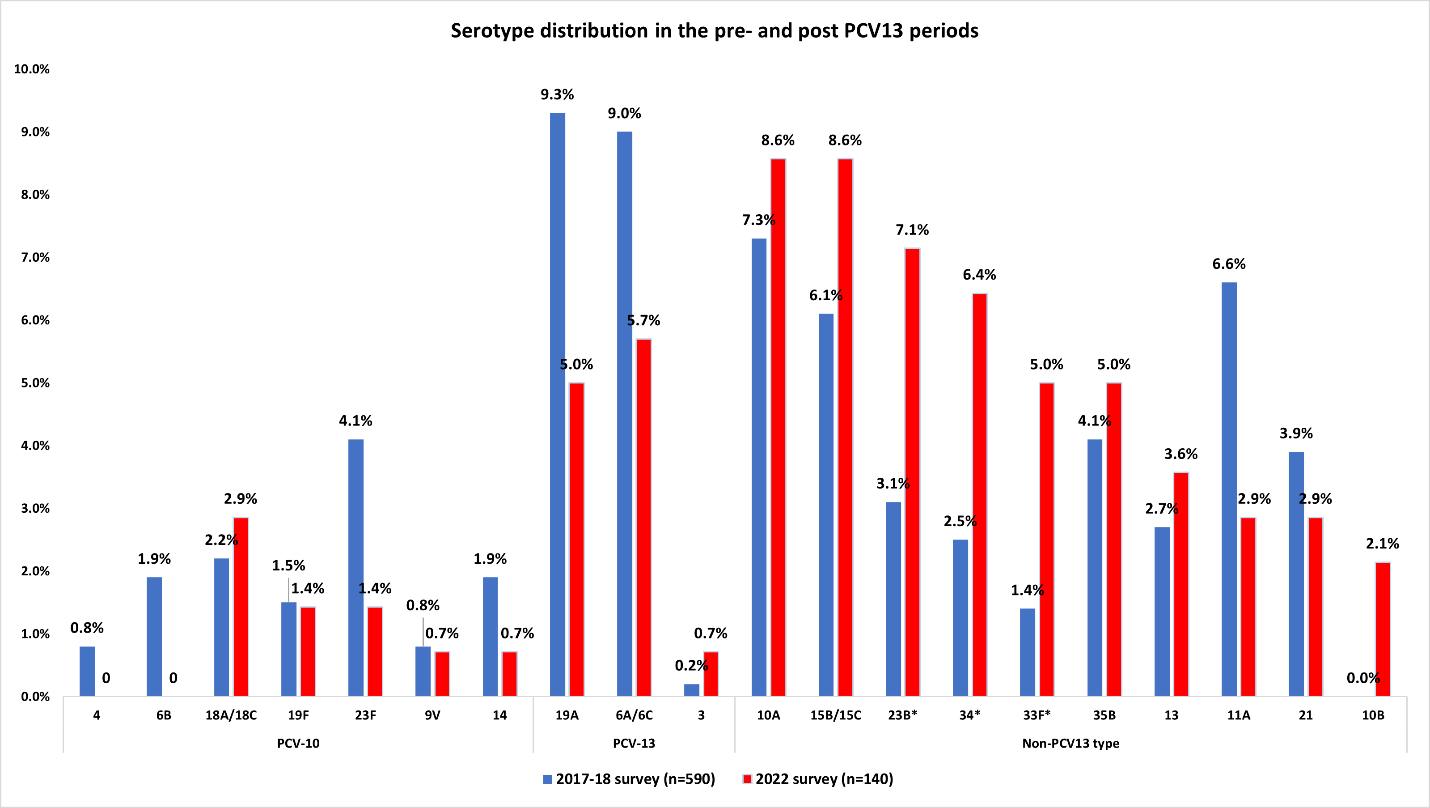

Supplement: Supplementary file 1 — Supplementary material 1- Serotype distribution in the pre- and post PCV13 period [file mmc1.docx]
