# Supplementary material for: Pneumococcal carriage and changes in serotype distribution post- PCV13 introduction in children in Matiari, Pakistan
Source: Vaccine. 2024 Oct 3;42(23):None. doi: 10.1016/j.vaccine.2024.126238 (PMC11413484; doi:10.1016/j.vaccine.2024.126238)
Supplement: Supplementary file 2 — Supplementary material 2- Antimicrobial resistance patterns [file mmc2.docx]

**Table S1. Antimicrobial resistance patterns among carriage isolates in Pakistan**

| **Antibiotics** | **2022 (N= 140)** | | | **2017-18 (N= 590)^*^** | | |
| --- | --- | --- | --- | --- | --- | --- |
|  | **Non-PCV13 serotypes** | **PCV13 serotypes** | **Total** | **Non-PCV10 serotypes** | **PCV10 serotypes** | **Total** |
|  | **N=114 (81·4%)** | **N=26 (18·6%)** |  | **N=512 (86·8%)** | **N=78 (13·2%)** |  |
| **Penicillin (meningitis)** | 92 (81·4%) | 21 (18·6%) | 113 (80·7%) | - | - | - |
| **Penicillin (non-meningitis)** | 0 (0%) | 0 (0%) | 0 | 0 (0%) | 0 (0%) | 0 |
| **Erythromycin** | 46 (70·8%) | 19 (29·2%) | 65 (46·4%) | 149 (81·9%) | 33 (18·1%) | 182 (30·8%) |
| **Tetracycline** | 54 (70·1%) | 23 (29·9%) | 77 (55%) | 201 (81·4%) | 46 (18·6%) | 247 (41·9%) |
| **Cotrimoxazole** | 100 (80·6%) | 24 (19·4%) | 124 (88·6%) | 481 (86·7%) | 74 (13·3%) | 555 (94·0%) |
| **Chloramphenicol** | 2 (100·00%) | 0 ( 0·00%) | 2 (1·4%) | 14 (93·3%) | 1 ( 6·7%) | 15 (2·5%) |

*The data is from a pre-PCV13 survey by Nisar et al, the results are based on culture and antimicrobial susceptibility testing by standard Kirby-Bauer disk-diffusion method on Mueller-Hinton Agar (MHA) with 5% sheep blood agar as per Clinical & Laboratory Standards Institute (CLSI) recommendations **^(10)^**.
